# Balancing burdens of infection control: Norwegian district medical officers’ ethical challenges during the COVID-19 pandemic

**DOI:** 10.1186/s12913-023-09573-7

**Published:** 2023-06-07

**Authors:** Linn Brøderud, Reidar Pedersen, Morten Magelssen

**Affiliations:** 1 Centre for Medical Ethics, Institute of Health and Society, University of Oslo, Pb. 1130 Blindern, Oslo, N-0318 Norway; 2 MF Norwegian School of Theology, Religion and Society Oslo, Oslo, Norway

**Keywords:** COVID-19, District medical officer, Municipal health care services, Norway, Public health ethics, Qualitative interview study

## Abstract

**Background:**

In several countries, district medical officers (DMOs) are public health experts with duties including infection control measures. The Norwegian DMOs have been key actors in the local handling of the COVID-19 pandemic.

**Methods:**

The aim of the study was to explore the ethical challenges experienced by Norwegian DMOs during the COVID-19 pandemic, and how the DMOs have handled these challenges. 15 in-depth individual research interviews were performed and analyzed with a manifest approach.

**Results:**

Norwegian DMOs have had to handle a large range of significant ethical problems during the COVID-19 pandemic. Often, a common denominator has been the need to balance burdens of the contagion control measures for different individuals and groups. In another large set of issues, the challenge was to achieve a balance between safety understood as effective contagion prevention on the one hand, and freedom, autonomy and quality of life for the same individuals on the other.

**Conclusions:**

The DMOs have a central role in the municipality’s handling of the pandemic, and they wield significant influence. Thus, there is a need for support in decision-making, both from national authorities and regulations, and from discussions with colleagues.

## Background

The COVID-19 pandemic has brought tremendous challenges to healthcare services and societies all over the world. Well-functioning public health services at a local level are probably vital to deal effectively with pandemics. In several countries, *district medical officers* (DMOs) are key experts in the public health services, with special obligations concerning infection control both in health care specifically and in the community in general. To handle the pandemic and safeguard public health, imposing restrictions on individual freedom and rights was often deemed necessary [1]. However, imposing such restrictions may give rise to value conflicts and ethical challenges. An *ethical challenge* is a situation where norms or values conflicts, and there is doubt, uncertainty or disagreement about what is right or good [2]. (*Norms* are rules that guide action and can take various forms, such as professional, social, moral, and legal. *Values* are something deemed good and desirable.) The aim of this study was to characterize ethical challenges concerning infection control experienced by Norwegian DMOs during the pandemic. Knowledge of such ethical challenges is important in itself and might aid decision-making in the future [3]. However, a study of experienced ethical challenges also provides a particular perspective on the pandemic. This perspective might enable us to learn something about the pandemic and its impact, and about the particular role of the DMOs.

### The COVID-19 pandemic in Norway

As of 9 April 2023, there have been 5369 registered COVID-19-related deaths in Norway. The average age of the dead is 83 years and 53% are men [4]. Compared to other countries, COVID-induced burdens of disease and death have been relatively light in Norway [5, 6]. Norway is among the Western countries with the lowest number of hospitalized COVID patients compared to the size of the population [5], the lowest COVID mortality and where the economy is least affected [6]. Among the likely causes of Norway’s relative success in handling the pandemic is a well-developed publicly funded health care system where the municipalities have the main responsibility for infection control and public health measures [6]. In addition, a primary care system with general practitioners has been argued to contribute to reduced mortality and hospital admission rates in general [7]. A strong primary health care service contributes positively to public health [6, 8]. Knowledge of local conditions is important when the municipalities are to trace infection spread, assess severity of outbreaks and implement infection control measures. A recent report considered the municipalities the backbone in Norway’s handling of the pandemic [6]. In this respect, Norway’s municipalities have been judged competent and able to quickly adapt to new tasks [9]. In a Scandinavian comparative perspective, the Danish municipalities have received a similar evaluation; in contrast to Sweden, which has a system and structure of care for the elderly that differs from Norway and Denmark in several respects [9, 10].

### Organization, legislation, and the role of the district medical officer in the pandemic

On the national level in Norway, three bodies are key actors in infection control. First, the Ministry of Health and Care Services has the overall responsibility for infection control in Norway. Second, the Directorate of Health shall execute the Ministry’s political decisions. Third, the Norwegian Institute of Public Health oversees epidemiological surveillance and scientific advice. The primary responsibility to implement the national infection control policies and to handle the pandemic in the communities, however, has been assigned to the 356 municipalities.

The Municipal Health Services Act § 5–5 states that every municipality must employ at least one DMO [11]. The municipalities vary in size, and some have employed several DMOs, whereas some have only one who might serve several municipalities simultaneously. Many DMOs have part-time positions in combination with work as a general practitioner [11, 12]. The DMO is to be the municipality’s medical advisor [13], with a particular responsibility for infection control and detection. § 7 − 2 in the Infection Control Act gives an overview of the DMO’s tasks related to the latter responsibility. According to the act the DMO must plan and lead the municipality’s efforts for infection control, have an overview of the epidemiological situation, and inform and advice municipal health professionals and institutions and the local population.

The municipalities have had extensive responsibilities during the pandemic, both as exercisers of authority and as service providers. For instance, contact tracing and vaccination are organized by each municipality. The municipalities would both communicate and enact national measures, develop and enact their own local measures, and give advice to their local population [6].

Through the Infection Control Act [14], both the national authorities and the municipalities have the authority to instigate comprehensive and intrusive measures to counteract infectious diseases. Measures must have a clear medical justification and be judged necessary and apt on an overall assessment. Voluntariness should be the norm.

The municipalities have the authority to establish stricter (but not milder) measures than the present national measures. In accordance with § 4 − 1, the municipalities can decide local regulations on, for instance, the closure of businesses or other restrictions regarding freedom of movement, depending on the local epidemic situation. The competence to make decisions pursuant to § 4 − 1 has initially been given to the municipal council. However, DMOs are often involved as medical advisors with special competence in public health work, including infection control, and have also been given authority to make decisions on their own, especially in emergency situations [6]. A study found that 62% of the local resolutions made between March and November 2020 were made by a DMO in the Norwegian municipalities [15]. Another qualitative study on the role of the DMOs during the pandemic found that the DMOs were key actors in crisis management whose knowledge was sought and valued by the municipal management [16].

Little empirical research on DMOs’ experienced ethical challenges has yet been published [17]. Most previous Norwegian research on ethical challenges during the COVID-19 pandemic has focused on the hospital and nursing home contexts [18–21]. One study examined challenges experienced by nine nursing home doctors [18]. Among the challenges were deciding on visiting restrictions and the use of coercion for testing and isolation.

## Methods

### Aim and design

The aim of the study was to explore the ethical challenges that Norwegian DMOs have experienced during the COVID-19 pandemic and how they have handled these challenges. A qualitative approach with in-depth individual interviews was therefore chosen.

### Recruitment

We included a diverse set of municipalities in order to capture a wide range of experienced challenges. We sought geographical spread as well as diversity in participant gender and age. This was by and large achieved. A total of 44 DMOs were invited successively until 15 interviews had been conducted. At this stage saturation was deemed to have been achieved. The first contact was by email. Potential informants were sent invitations, information and a consent letter.

### Participants

Of the 15 participants the average age was 52. Eight of the informants were women. Mean years of experience as a DMO was 10 years. Eight of the DMOs were the sole DMOs in their respective municipalities. Some of the informants split their time between GP and DMO work; that is, their position as a DMO was only part-time. The DMOs in the study represent in total twenty-five municipalities (several served more than one municipality), ranging from small to large. Ten DMOs were from Eastern Norway, three from Western Norway and two from Middle Norway.

### Interviews

A written interview guide with topics to be explored was employed. The interview guide was revised in light of a pilot interview with one DMO. In the interviews, the intention was to let the informants spend the most time on what they themselves believed had been most important. The interviews were conducted individually using the digital video conferencing software Zoom during February to May 2021. The interviews lasted about 45–60 min and were recorded and transcribed.

### Thematic analysis

A qualitative content analysis in line with recommendations from Graneheim and Lundman [22] was performed. The analysis took a manifest approach, which means that interpretation stayed close to the text and the expressed meaning. Involving several steps, the method began by reading through the interviews several times to obtain a sense of the whole. The text was then divided into meaning units that were condensed and labelled with a code. The various codes were compared based on differences and similarities and sorted into main themes, which again were sorted into categories and sub-categories for each theme. These were then discussed by the researchers and revised. The process involves a back-and-forth movement between the whole and parts of the text.

## Results

The analysis resulted in two main themes which were further divided into categories (Table 1). The first theme is the direct answer to the study’s research question concerning the nature of the ethical challenges Norwegian DMOs have experienced during the pandemic. The second theme concerned the DMOs’ role in ethical decision-making.

**Table 1:** Main themes and categories (sub-categories not shown)

Main theme	Categories
Ethical challenges the DMOs have experienced during the pandemic	Relative neglect of the interests of vulnerable groups
	Balancing the need for infection control measures with other interests and negative consequences
	Infection control versus confidentiality and privacy
	Challenges in fair resource allocation
Handling the ethical challenges	Between strict guidelines and discretionary power
	Collegial support within and outside the municipality
	Limited ethical reflection

### Theme 1. Ethical challenges the DMOs have experienced during the pandemic

Consistently, the informants described the need to achieve effective infection control for the sake of the common good as a predominant value. This value then had to be balanced with different conflicting values. Measures instigated led to burdens on certain vulnerable groups, as detailed in the first category.

#### Relative neglect of the interests of vulnerable groups

*Children and young people.* A majority of the informants pointed to children and young people as one of the groups most affected by the national objective of limiting social contact. Lockdowns involved closure of schools and kindergartens and organized sports and other activities. The informants argued that this had enormous consequences for children’s and young people’s social life, more so than for other age groups. During the first lockdown, children from vulnerable homes, now spending more time at home, were thought to be particularly affected. One informant stated that this constituted a problem that the municipal health care services did not have the resources to follow up. To enforce social restrictions was described as creating a significant dilemma, considering children and young people themselves were at low risk for serious illness and death by COVID-19:

[…] it was almost as if it was based on such an idea ‘for the greater good’, that society demanded, but some had greater consequences than others. For example, children from vulnerable homes. […] but the children [themselves] were not protected by this. We knew quite early on that children didn’t have risk of a serious disease course and death by the COVID-19 virus. Children were being kept home from school simply to protect society.

Several mentioned setting the level of measures in schools and kindergartens as ethically challenging, also due to uncertainty about its efficacy for curtailing spread of contagion, considerable time pressure in decision-making and pressure from external actors who advocated for either lower or higher measure levels. One added that the level of measure was thought to be mainly a professional or medical assessment of what effective infection control requires, yet ethically speaking, the special needs of children should also be taken into account. Some felt that the interests of children should have been given more weight by the municipal management. Many teachers, on the other hand – not being prioritized for vaccines – felt exposed and were afraid of getting infected, and parents held a variety of views.

*Elderly and nursing home residents.* Daytime activity centers were reduced or closed, and strict visitation rules were put in place in nursing homes to protect old and frail residents with high mortality risk if infected. This was described by some DMOs as a situation where they made the choice on behalf of the patients to protect them from COVID-19, but at the same time deprived patients of the opportunity to be with their loved ones in the last part of their life:

The sacred situation where one has to say goodbye… if we should be unlucky to let the devil [i.e., the virus] into the institution… it’s such an ethical dilemma; what’s the least bad option of those two […]. It’s a situation where none of the choices are good. One must choose the one that takes care of the majority. But I have no good feeling about this. I think we have made some wrong choices. I think we have been too scared.

Another informant said, *“We have old people who would say that they are not that afraid of infection and they will die of something anyway, but we [the health care providers] have an incredible pressure to not expose this patient group”.*

However, some stated that their municipality went to further lengths to maintain social contact for the dying than for other nursing home residents, such as those with impaired cognition who might handle isolation poorly.

I think we probably could have done more to facilitate for individuals [with dementia], but it is difficult to achieve a personalized approach when you have to follow some guidelines. They are too coarse-grained.

Visitation restrictions were justified in different ways. One said that they had no other effective infection control measure than to keep visitors out. Some expressed concerns regarding potential sick leave among staff if there was to be an outbreak in institutions. Another DMO explained that they considered COVID-19 as a potentially severe plight for the patient often involving more suffering than the patient’s chronic illnesses. The dilemma of visitation restriction was further complicated by the fact that the nursing home residents often have a short life expectancy.

In institutions, infection among the cognitively impaired who wandered about gave rise to a dilemma: should one protect fellow residents by moving the patient, e.g., to a hospital ward, or should one prioritize the patient’s need for familiar surroundings?

*People with learning disabilities.* This group often had their activities and adapted work restricted or closed, and DMOs often found it difficult to decide on when was the proper time to reopen. Restrictions were argued to be problematic, as boredom, loneliness and more acting-out behavior ensued. In some places, activities were closed down because of care workers’ fear of infection or uncertainty whether this group could handle basic infection control measures. One informant argued that the autonomy for this group “goes under the radar”.

*Cultural, religious and foreign language groups.* Providing information and follow-up to some groups was perceived as challenging because of the lack of a common language and understanding, differences in cultural and religious beliefs, trust in authorities and information sources, and compliance with guidance. DMOs stated that they had to approach some groups differently. Several also pointed out that public updates on contagion numbers, e.g., to local newspapers, ran the risk of stigmatizing certain groups such as migrant workers, religious congregations and ethnic minorities.

#### Balancing the need for infection control measures with other interests and negative consequences

*Closing down of municipal health center activities.* During the pandemic many municipal healthcare workers were reallocated from their original responsibilities to pandemic-related tasks. This was not without problems. In particular, informants pointed to challenges created by the reallocation of public health nurses from their primary tasks to infection tracing. The needs of the nurses’ primary target groups (e.g., new mothers, schoolchildren) were then for a period not met. In some municipalities, nurses who routinely conducted visits in patients’ homes were quick to suspend these activities because of fear of spreading infection and themselves becoming infected. It was argued that this shutdown was an overreaction, due to uncertainty about how the virus spread. One DMO reflected upon the challenge of how health care workers’ own fear might compromise services for vulnerable people. Here, health authorities’ communication to professionals about risks and measures was described as too vague at times, creating room for misunderstandings.

*Higher threshold for patients to see their GPs.* A general recommendation was issued to avoid unnecessary requests to health services. Several informants were concerned that specific patient groups, such as those with mild or moderate mental illness, had received poorer care than before the pandemic, and that some patient groups have simply refrained from seeing their GPs in spite of medical needs. Long-term consequences for these individuals were thought potentially to be greater than initially recognized.

*Closing down of gyms and organized physical activities.* Gyms and organized physical activities were closed down at times during the pandemic in order to reduce contagion. Deciding whether or not to close down, and the consequences of such measures, were recurring themes for several of the DMOs. One informant said that it has been challenging to maintain infection control, especially in the unattended gyms where restrictions were often not observed. This often led to decisions to close the doors to everyone, including those with rehabilitation needs. The DMO acknowledged that the gyms are important to many, both mentally and physically, for some people being their only social arena.

Many activities and sports were suspended because it was considered difficult to keep distance and avoid physical contact. However, one DMO said that they gradually had deviated from the national recommendations and reopened activities for vulnerable groups, thus tolerating the increased risk of infection that ensued.

*Advising against travel to neighboring municipalities.* One informant mentioned discouraging travel between the neighboring municipalities because of the neighbors’ high infection rates and the fear of the infection spreading due to this. The DMO described it as challenging to implement this measure because it would affect many people’s way of living, trade, and in particular, the relationship between the municipalities’ managements.

*Vaccination without patient consent.* According to some DMOs, some nursing home residents lacking competence to consent were vaccinated without an individual appraisal of benefits and risks. One DMO added that everything had to happen rather quickly, and the nursing home doctors often could be young and lacking experience and knowledge about relevant legislation. The use of coercion when incompetent patients resist vaccination was also raised as an ethical challenge. One informant stated that patients were vaccinated without being given an actual choice. Patients did not receive information about consequences, quality of life or life expectancy. Significantly, Norwegian legislation does not authorize coercive vaccination. Sometimes next of kin pushed for the patient to be vaccinated. One stated that “relatives will, of course, perhaps without even knowing it, promote their own view more than the patient’s view, which is the view they are actually supposed to promote.” In institutions, a complicating factor was that refraining from vaccinating might increase risk of contagion and thus entail a danger to other residents. One informant characterized this dilemma thus, “it is the classic individual’s right against the group’s right.”

*Quarantine and isolation* of individuals were among the most drastic infection control measures undertaken. Quarantining people was described by some informants as “severely intrusive”. Some wondered whether they at times had been too tough, for instance by going into people’s homes and ordering them to quarantine hotels when not following the instructions. Several said that it had been ethically challenging whether to report intentional violations of quarantine or isolation requirements to the police. It was described as frustrating for the infection detection team to witness people risking infecting others. However, informing the police might lead to people not being forthright in providing information, thus impairing infection tracing.

Another informant experienced an outbreak of COVID-19 in a housing community for drug users and in sheltered housing, where one used authority and force to detain those who would not respect the quarantine and isolation rules. This was described as a situation “that left a bad taste”, but where infection control was perceived as overriding autonomy in importance.

*Pressure for stricter measures.* Several DMOs described experiencing pressure from different stakeholders for stricter infection control measures. For instance, in schools and kindergartens, teachers and sometimes parents might want the institutions to close down for a period of time. Some admitted that the pressure could be a contributory factor in their own decisions about measures, especially if the situation was at a tipping point where they were unsure about the best course of action. A couple of informants problematized that infection and death rates were being reported daily in the media but without context. From their perspective, the fact that those at risk for a fatal disease course were mainly the elderly with comorbidity did not appear to come across to the public. Thus, an exaggerated fear of infection in the population appeared to contribute to pressure on DMOs to instigate stricter measures and a desire to see infection rates going down towards zero. Again, however, measures were likely to have adverse consequences for people, some of whom were themselves at little risk: “*Having deaths [by COVID-19] in the municipality was a big thing and was considered as such a loss of quality for the municipalities, as a kind of shame […], so we were quite eager to prevent any deaths.”*

Several argued that during the pandemic one must tolerate some level of risk of infection spread; otherwise one would at all times have severe measures that would constitute unjustified restrictions on people at low risk. However, this was perceived as all in all a balancing act, and several pointed to an actual and justified fear of losing control of the epidemic. Several problematized the balance between the burden of measures versus the positive effects. Proportionality was challenging to assess; the DMOs had to find adequate medical justifications for the measures they wanted to implement. The measures had to be both effective and proportionate in relation to the disadvantages they entailed.

#### Infection control versus confidentiality and privacy

Informants spoke of the challenge of balancing the duty of confidentiality with the need to share information to enable infection tracing. Fundamentally, the DMOs have an obligation of confidentiality, which they appeared to strive to maintain. Yet by the Infection Control Act they are authorized to breach confidentiality by for instance giving up names if required. It was particularly challenging to maintain confidentiality in infection tracing when, for instance, an infected person potentially could have had close contact with a health care worker and the infection detection team had to give up names in order to quickly trace pathways; or due to people often requiring a reason (i.e., the name of a close contact) for why they had to be quarantined. This led to names being shared, and this could lead to situations where, for instance, the DMO gave up a name to an employer and the name was then spread to all employees at the infected person’s workplace.

Several stated that information sharing in the media was challenging. DMOs were expected to update local media on infection outbreaks. Such transparency was also in line with municipal policy. However, especially in smaller communities, information sharing often made it possible to identify individuals or groups, potentially leading to risks of stigmatization or other repercussions. In addition, some informants stated that not everyone in the municipal management were used to handling the duty of confidentiality. In some situations, they would share too much information in the media. One informant reflected on these practices:

Consideration for the majority becomes decisive […] actually, an almost somewhat unusual openness about these things, and I experienced it as a kind of requirement from the municipality management, to be able to plan further what measures were necessary in terms of infection control. […] the confidentiality part… The name was never given, but it was recognizable in some cases, that’s quite obvious. It’s probably interesting to look further at the issue of confidentiality.

#### Challenges in fair resource allocation

The informants had experienced several ethical challenges concerning priority setting or fair resource allocation, particularly concerning scarce resources such as vaccines, hospital beds and infection control equipment.

*Vaccination.* A national vaccine priority setting guideline was published before vaccines became available in large quantities. Although almost every informant expressed satisfaction with this guideline, it did not prevent all ethical problems. Informants had experienced several ethical challenges concerning vaccination priority. Some DMOs believed that certain patient groups should have been given higher priority for vaccination, and that the national guideline was too coarse-grained.

The DMOs had different experiences and opinions concerning their own room for discretion in vaccine prioritization. Some chose to diverge from the national guidelines and gave health professionals higher priority at the expense of the sick and elderly, because of the need to safeguard the health service. Several informants had received numerous requests for higher prioritization, which had been challenging because many requests were backed by good reasons, both from individual patients, patient groups and physicians. Some of the DMOs chose to comply with some of the requests, thus for instance vaccinating a fifty-year-old cancer patient in palliative care before an eighty-year-old person. Another DMO invoked considerations of justice, arguing that when others made these kinds of discretionary assessments in specific cases in their municipalities, that in turn led to increased expectations, pressure and thus challenges for other municipalities. Several argued the importance of following national guidelines to avoid a more individual priority setting and the dilemmas that ensued.

Some stated that they had room for discretion in internal prioritizing of health care workers, but it was described as challenging and even uncomfortable to create a priority list. Questions that arose were, for instance, who should be defined as a health care worker? Which health care workers should be vaccinated first? Ought some groups of health care workers to be prioritized above patients? Some informants had seen situations where health managers and mercantile personnel were vaccinated before they ought to have been.

*Hospitalization.* Several found it ethically challenging to create advance plans and priorities for hospitalization of institutional- and GP patients. One informant reflected on the values at stake, arguing that patient autonomy was a neglected value in the sense that patients’ own preferences were not elicited or given weight. Another DMO had challenged local GPs to set priorities for hospitalization, because it was seen as essential that every admission was based on an individual evaluation. One DMO problematized the message communicated to the public that hospitals must not be overloaded. This was a balancing act where one did not want to use up hospital capacity for those who could receive adequate care in the municipality. On the other hand, there was a concern that this message led people who *ought* to have been treated at the hospital not to seek help. Informants claimed that an observed decline in cardiovascular incidents corroborates this concern.

*Infection control equipment.* In the early stages of the pandemic infection control equipment was a scarce resource, particularly in municipalities. One informant claimed that they received clear signals from the national authorities to be restrictive in using equipment. From an early stage it was deemed that the bulk of the municipality’s equipment had to be allocated to elderly care, meaning other services did not get any. The informant received pressure from other services who felt neglected, such as assisted living facilities for people with learning disabilities.

### Theme 2. Handling the ethical challenges

Concerning the handling of ethical challenges recurrent topics in the interviews were the balance between guidelines and discretionary power; the significance of collegial support; and the relative absence of explicit ethical reflection.

The DMOs’ degree of involvement in and responsibility for decision-making varied. They were least involved in situations where they were mostly communicating and passing on decisions made by others, such as national guidelines and advice. At the other end of the scale, they were most involved when being directly responsible for making decisions on the basis of the Infection Control Act.

Some of the DMOs saw themselves as someone who should balance all considerations and make explicit any consequences that were not apparent in the short term. Some argued that their role and training involved considering the interests of the population more than the interests of the individual, yet also giving specific attention to the needs of the most vulnerable. Some claimed that they generally took a more liberal position – typically arguing *against* more restrictive measures – in deliberations together with institutional and municipal leadership: “from being the one who should be strict in the beginning, I quickly became the one who tries to slow down [e.g., concerning visiting restrictions in nursing homes].“

#### Between strict guidelines and discretionary power

The majority had experienced a great deal of trust within the municipality management, and as a consequence of this, significant discretionary power was given them. In general, the DMOs seemed comfortable with the responsibility that came with this power, but some did also express feeling burdened by or uncomfortable with the discretionary assessments they had to make.

Detailed national guidelines were not available at the beginning of the pandemic. Several mentioned the lack of such guidelines as problematic. At that time, the DMOs found themselves “in a vacuum” characterized by insecurity and a fear of doing too little to curb the epidemic locally, when at the same time the prospect of implementing strict measures appeared drastic. It was also described as stressful to be the one who had to burden people with restrictions on their daily life. In turn, it was described as a relief when national guidelines were finally presented. This reduced the need for discretionary assessments: *“it’s a kind of assurance to follow the national guidelines to prevent us from getting into situations where you have to say, ‘no, I consider you not to be as worthy as he is’ [i.e., prioritizing between the needs of different citizens].”* However, many mentioned the communication from the national level as leading to problems. For instance, information was often given orally at national press conferences announced at short notice, and only published in writing several days later. This created a vacuum where DMOs had to interpret the guidelines. This had negative consequences such as different practices between municipalities and secondarily public dissatisfaction.

Among informants, opinions and perceptions were divided concerning discretionary assessment and decision-making: concerning how large their scope for discretion was; whether large discretionary space was desirable; and which main areas and types of decisions it affected. Several stated that the possibility of making discretionary assessments was regulated by the infection situation. A majority preferred clear rules and frameworks to counteract too large differences in practice.

#### Collegial support within and outside the municipality

Almost every DMO said that they have been quite dependent on collegial support during the pandemic. Some stated that the role had been lonely at times, especially for those who were the municipality’s only DMO. Many had been on more or less continuous duty at the time of the interview, and leisure time had been scant. They had found support in various places, such as in neighboring DMOs or other DMOs throughout the country who had experienced the same challenges. Some municipalities had already prior to the pandemic established inter-municipal cooperation where the DMOs had continuous discussions between them. Some municipalities had several DMOs employed, and those who worked with local colleagues argued that this had been a crucial support. Some found support in the municipality management and argued that it was productive to discuss difficult matters with others who had a different mindset or approach. Almost all emphasized that the support received from The Norwegian Institute of Public Health was essential. A few mentioned the benefit of a close cooperation with the County Governor (no.: “Statsforvalteren”), and added that they had received answers rather quickly when needed.

#### Limited ethical reflection

To what extent ethical reflection functioned as an active tool for the DMOs’ decision-making seems to have varied. Some said that even though they have not called it ‘ethics’, many of the issues they have grappled with the most have been ethical in nature. For some, ethical reflection functioned as a filter and a guide when facing dilemmas. For others, it may have been more difficult to distinguish the ethical challenges from professional, legal, and practical challenges. Having control of the infection situation was the main focus, and due to this, ethical reflection had for many been deprioritized. Several stated that there had not been time to take all considerations into account, “It has been like being on an eternal run that never stops”.

When asked how they had reached decisions in ethical problems during the pandemic, several answered that they first of all looked at the legislation, regulations and the guidelines in accordance with the infection situation. Some sought consultations with the municipal management or involved the relevant parties, and some pointed to the explicit weighing of the consequences of various measures. Some argued that the specialization in community medicine that they have undergone provides solid foundations for ethical decision-making. Several of the older DMOs claimed that age and experience are relevant in dealing with these ethical dilemmas.

Some of the informants had ideas for how the handling of ethical challenges could have been improved. They mentioned more time for experience sharing, a professional ethicist to turn to for dialogue, a guidance on how to use discretion and how to handle the media and a system that ensures collegial support in ethical considerations. Several informants argued that ethical reflection in the municipal health service, as for instance as an active tool in risk and vulnerability analyzes, should be promoted more. One informant added, “I think it will cost us dearly not to have had ethical reflection along the way”.

## Discussion

In general, the DMOs have supplied rich descriptions of a host of ethical problems. In what follows we comment on some overarching findings.

### Balancing burdens

A common denominator for several of the ethical problems was that these problems at their core were issues about fair distribution and balancing of burdens. This is an instance of the “balancing act” which arguably is intrinsic to public health work [23]. The closure or restriction of access to, e.g., schools, gyms and public facilities were instigated in order to lower the general level of contagion risk in the local society. Here, the utility of the measures was low for the individuals affected.

However, for another main category of ethical problems there was significant utility for individuals. Here, the issue was to achieve a balance between safety understood as effective contagion prevention on the one hand, and freedom, autonomy and quality of life for the same individuals on the other. Here, access restriction to nursing home was a prime example.

Apparently, several «classical» ethical challenges (e.g., end-of-life decision making, patient autonomy, priority setting and confidentiality) have been compounded or become more complex because the issue of contagion control has entered as another complicating factor which must be taken into consideration. For instance, this was the case in the heightened need for advance care planning for nursing home residents, in vaccination without a valid consent, and in providing information to the media whilst protecting confidentiality.

### Uneven distribution of burdens and utility, and lack of knowledge base

The findings corroborate other reports and contentions in that burdens and utility from contagion prevention measures have been unevenly distributed [5, 24]. Both economic and non-economic harmful effects have particularly affected vulnerable groups in society, and inequality in the risk of infection, severity of disease and economic and social consequences of the pandemic between different groups can increase social inequality in health [24].

Due to COVID-19 causing significantly more mortality and morbidity in the elderly and in patients with chronic diseases, other groups less at risk have carried large burdens from the measures without receiving a proportional utility in the form of risk reduction. The Norwegian Corona Commission Report argued that strict infection control measures and a reduction in access to municipal health care services have led to increased loneliness and isolation for the oldest in society and for users with complex needs. This can lead to impaired health, reduce the ability to function and is even associated with increased mortality and a risk of other diseases for the elderly [24].

The results show that the DMOs had to prioritize and instigate measures without a solid knowledge base about effectiveness and side-effects of the measures, especially in the beginning. The Public Health Report for 2021 argued that in Norway, a large part of the health consequences was likely to be caused by the infection control measures themselves [5]. For instance, the harm caused to schoolchildren by school closures was a significant worry for many of the DMOs, yet one that could hardly be quantified at the time decisions were made [25, 26] [5, 27–29]. Norway closed all primary-, lower secondary- and upper secondary schools in March 2020 for about 6–8 weeks. Also after this, many pupils’ everyday lives have been characterized by unpredictability, restrictions and home schooling in periods [5].

In the aftermath of the pandemic, it is important to examine whether priorities in contagion prevention measures were apt and fair, or whether – as seems likely – certain groups have carried a disproportionate share of the burdens. Our study is not designed to provide such an examination. The study does, however, provide interesting clues concerning where to look, in the form of the first-hand reports of central actors in the municipalities’ infection control apparatus. Among the main groups that they point to as potential victims of the infection control measures are, in addition to schoolchildren, patients with mental and other chronic conditions reliant on close GP follow-up; the elderly; people with intellectual disabilities; minorities; and new mothers.

### Discretion and decision-making support

The DMOs saw the ethical issues they have faced during the pandemic as value conflicts where different factors must be weighed. Again, «balancing burdens» describes well what the handling of ethical problems involved from the perspective of the DMOs. In general, the DMOs perceived themselves as competent to perform this ethical «balancing act»; although decisions could be taxing, the DMOs perceived the decision-making as something firmly belonging to their professional role [30]. At the same time, they valued very highly the guidance from the Norwegian Institute of Public Health, which has a national coordinating role for the DMOs, and the informal discussion with colleagues locally or regionally.

The DMOs appear to have had significant space for discretionary assessment and decision-making during the pandemic. This resonates with a study of the DMO role which emphasized their wide autonomy [30]. In general, a large space for discretion allows for «ethical tailoring» of proper solutions based on the exact conditions there and then. At the same time, discretion can create divergent practices and (the perception of) injustice. As also some of the informants emphasized, when made public such divergent practices can also lead to societal unrest and reduced trust in officials’ handling of the pandemic.

### Strengths and limitations

We have interviewed experienced public health experts with duties that include infection control measures and received in-depth accounts about the challenges they have faced during a pandemic. We have included participants from small, medium and large municipalities, as well as rural and urban municipalities. We sought to include municipalities with a moderate to high infection pressure at the time of recruitment, due to our assumption that ethical challenges arose as the infection rates increased. A selection bias cannot be excluded. Unfortunately, the regions of Southern and Northern Norway were not represented in the study as no DMOs from these regions accepted the invitation to participate. Interviews with DMOs from these regions might conceivably have given other results.

Transferability of the results to other countries is limited by the fact that the role of the Norwegian DMOs is rather specific to the Norwegian healthcare system. However, the study has highlighted the “balancing acts” of specific values and interests that must be made by those responsible for public health in the time of a pandemic.

## Conclusion

Norwegian DMOs have had to handle a large range of significant ethical problems or value conflicts during the COVID-19 pandemic. A common denominator for many of these ethical problems has been the need to balance burdens of the contagion control measures for different individuals and groups. The DMOs have a central role in the municipality’s handling of the pandemic, and they wield significant influence. Thus, there is a need for support in decision-making, both from national authorities and regulations, and from discussions with colleagues. It could be helpful to develop and test ethics support services tailored to the challenges of public health ethics nationally and locally.

## Data Availability

We are unable to share the data (transcripts) due to the conditions in the research ethics approval: the participants might be identified from the transcripts and they have not consented to sharing of these data. Any requests should be directed to the corresponding author.

## List of abbreviations

DMO: District medical officer
